# Post Meal Exercise May Lead to Transient Hypoglycemia Irrespective of Glycemic Status in Humans

**DOI:** 10.3389/fendo.2020.00578

**Published:** 2020-09-02

**Authors:** Jay W. Porter, Ryan J. Pettit-Mee, Sean T. Ready, Ying Liu, Guido Lastra, Anand Chockalingam, Nathan C. Winn, Laura Clart, Jill A. Kanaley

**Affiliations:** ^1^Department of Nutrition and Exercise Physiology, University of Missouri, Columbia, MO, United States; ^2^Department of Endocrinology, Internal Medicine, University of Missouri, Columbia, MO, United States; ^3^Department of Cardiology, University of Missouri, Columbia, MO, United States; ^4^Department of Molecular Physiology & Biophysics, Vanderbilt University, Nashville, TN, United States

**Keywords:** hypoglycemia, exercise, type 2 diabetes, glucagon, glucose

## Abstract

During exercise, there is coordination between various hormonal systems to ensure glucoregulation. This study examined if hypoglycemia occurs during moderate-intensity exercise in non-obese and obese individuals with and without type 2 diabetes (T2D). Eighteen non-obese, 18 obese, and 10 obese with T2D completed 2 study days that included a meal at 1,800 h followed by rest (NOEX) or exercise (PMEX; 45 min/55% of VO_2_ max 2 h post meal). Glucose, insulin, and glucagon concentrations were measured throughout this 5.5 h period. Subjects with T2D had elevated glucose responses to the meal on both study days, compared to non-obese and obese subjects (*P* < 0.05). During evening exercise (PMEX), subjects with T2D had a greater drop in glucose concentration (−98.4 ± 13.3 mg/dL) compared to obese (−44.8 ± 7.1 mg/dL) and non-obese (−39.3 ± 6.1 mg/dL; *P* < 0.01) subjects. Glucose levels decreased more so in females than males in both conditions (*P* < 0.01). Nadir glucose levels <70 mg/dL were observed in 33 subjects during NOEX and 39 subjects during PMEX. Obese males had a larger exercise-induced insulin drop than obese females (*P* = 0.01). During PMEX, peak glucagon concentrations were elevated compared to NOEX (*P* < 0.001). Male participants with T2D had an increased glucagon response during NOEX and PMEX compared to females (*P* < 0.01). In conclusion, in individuals with varying glucose tolerance, there is a dramatic drop in glucose levels during moderate-intensity exercise, despite appropriate insulin concentrations prior to exercise, and glucagon levels rising during exercise. Moderate-intensity exercise can result in low glucose concentrations (<60 mg/dL), and yet many of these individuals will be asymptomatic.

## Introduction

During exercise, as carbohydrate utilization increases and muscle glycogen stores begin to decline, potentially blood glucose levels can begin to drop. Low blood glucose levels can result in dizziness, confusion, nausea, headache, blurred vision, etc., all of which could impact exercise performance. Mammals, however, have evolved to prevent a precipitous decline in glucose concentrations via the counterregulatory hormones to keep the levels in a very tight range during rest and exercise (1).

If hypoglycemia does occur during exercise, it manifests when intense exercise is initiated shortly after the consumption of a high carbohydrate food or during prolonged exercise as fuel sources become depleted (2, 3). In the 1970's and 80's, considerable research was conducted examining the effects of exercise on glucose levels, particularly in prolonged exercise. Much of this work (4–6) focused on the impact of the pre-exercise meal prior to prolonged exercise to prevent hypoglycemia in moderate to well-trained individuals. Additionally, many of these studies were conducted following a 6–12 h fast, and studied effects of feeding 15–90 min before exercise. Few people, however, truly fast prior to exercise resulting in a postprandial state when initiating exercise. Early literature suggests that there is a risk of rebound hypoglycemia if exercise follows the meal too closely. Recently, Kondo et al. (6) demonstrated that transient hypoglycemia after pre-exercise carbohydrate ingestion (30 min prior) occurred in 7/16 subjects with values below 72 mg/dL, and 3 subjects were below 54 mg/dL. Moreover, they noted that individuals with an enhanced insulin response to the pre-exercise meal tended to be more prone to transient hypoglycemia in the fasted state.

Much of the previous work (5, 6) has shown hypoglycemia in well-trained individuals, usually in males, and in response to prolonged exercise. It is unclear if untrained individuals experience hypoglycemia, or if hypoglycemia is impacted by metabolic health. This project examined if hypoglycemia occurs during moderate-intensity exercise in the evening in non-obese and obese individuals with and without type 2 diabetes (T2D). Previous work by our group (7) showed that resistance exercise 45 min post dinner meal reduced glucose in T2Ds but had a rebound glucose response following exercise. Delaying an exercise session until after the dinner meal may provide an optimal time to improve glycemia prior to bedtime. We hypothesized that non-obese and obese individuals without T2D would have better counterregulatory control and thus would have tighter glucose control.

## Methods

### Subjects

Eighteen non-obese, 18 obese, and 10 obese+T2D, males and females (25–65 yrs. of age) were recruited and signed an informed consent approved by the University of Missouri Institutional Review Board. Body mass index (BMI) was between 30 and 45 kg/m^2^ for obese subjects and <25 kg/m^2^ for non-obese subjects. Subjects were weight stable for at least the prior 6 months, and non-smokers. All subjects also had a screening oral glucose tolerance test (OGTT). Non-obese subjects had fasting glucose <100 mg/dL and a 2 h glucose level <140 mg/dL. Subjects also had a screening exercise stress test. Women on oral contraceptives were tested in the pill phase. Pregnant women were excluded.

The T2D subjects were either diagnosed as T2D by their physician or documented fasting glucose levels >110 mg/dL for 5 of 7 days (8). Diabetic participants withheld medications for glucose control the night prior to and during the study day.

### Experimental Design

This study is part of a larger project in progress (clinicaltrials.gov, #NCT03019510). Following initial screening, subjects completed two study nights in a counter-balanced design; (1) no exercise (NOEX), and (2) evening exercise−2 h post dinner (PMEX). Study day arrival was ~1,700 h and standardized dinner meal consumption began at 1,800 h. Blood sampling was initiated from ~1,740 h until 0700 h, however this paper will focus on the first 5 h of sampling. On an exercise day, subjects exercised for 45 min at ~55% VO_2_ peak on the treadmill.

### Screening Day: Anthropometrics and Questionnaires

Height, weight, and waist circumference were measured. Fat mass, fat-free mass, and percent body fat were assessed using a Bod Pod (Life Measurements, Concord, CA) or DEXA (Horizon A, Hologic, Marlborough, MA). All subjects had a screening OGTT, and hematocrit (Hct) was measured. Further, questionnaires were completed on the screening day to determine their inclusion in the study, and included a health inventory (9) and sleep apnea (Berlin questionnaire) (10).

All subjects completed a peak oxygen consumption test (VO_2_ peak) on a treadmill. Prior to starting the test, electrodes were placed for EKG and heart rate measurements during the test. The ventilation, and percent oxygen and carbon dioxide were measured by True One 240 Metabolic Measurement Cart; ParvoMedics (Sandy, UT) and VO_2_ was calculated. The test began with 2 min of very slow walking (2 mph). Speed and incline of the treadmill were increased every 2 min until the subject reached volitional exhaustion (11). Following the exercise test, subjects actively cooled down and were monitored for 5–10 min until HR and BP returned to near baseline.

### Study Day

The evening prior to the study night, subjects were provided with a dinner meal (~600 kcal; 56.1% Carbohydrate, 21.5% Fat, and 22.4% Protein) as well as breakfast, snack, and lunch for the study day (Table 1). Subjects consumed the breakfast at ~0700 h and lunch at 1,200 h. Subjects then fasted, except for water for the remainder of the day. There was no exercise or alcohol consumption for the 24 h prior to the study day.

**Table 1:** Subject characteristics and meal composition for the non-obese, obese, and type 2 diabetic subjects.

	**Non-obese**	**Obese**	**T2D**
*N* (Male, Female)	18 (9,9)	18 (5,13)	10 (2,8)
Age (years)	45.7 ± 3.6	42.6 ± 3.5	53.9 ± 1.9
Height (cm)	168.5 ± 2.1	168.0 ± 2.2	168.9 ± 3.4
Weight (kg)	69.6 ± 2.3	95.8 ± 4.0^*^	96.3 ± 9.4^*^
BMI (kg/m^2^)	24.4 ± 0.5	33.8 ± 1.0^*^	33.9 ± 1.8^*^
Body Fat (%)	27.9 ± 2.3	44.3 ± 1.8^*^	42.7 ± 3.0^*^
Fasting Glucose (mg/dL)	83.8 ± 2.5^†^	84.5 ± 1.8^†^	131.9 ± 9.4
VO_2_ peak (mL/kg/min)	36.5 ± 1.9	26.8 ± 1.1^*^	23.7 ± 1.7^*^
**Breakfast, snack, and lunch**			
Calories (kcal)	914.7 ± 40.5	1048.6 ± 70.7	962.2 ± 96.4
Carbohydrate (%)	52.1 ± 0.0	55.0 ± 0.1	50.7 ± 0.0
Fat (%)	28.3 ± 0.1	29.9 ± 0.0	27.7 ± 0.0
Protein (%)	14.3 ± 0.0	15.1 ± 0.0	13.9 ± 0.0
**Study dinner**			
Calories (kcal)	718.6 ± 72.2	919.6 ± 38.3	794.0 ± 28.5
Carbohydrate (%)	37.9 ± 0.4	40.4 ± 0.3	36.9 ± 0.0
Fat (%)	33.1 ± 0.1	35.0 ± 0.1	32.3 ± 0.0
Protein (%)	23.7 ± 0.6	24.6 ± 0.5	23.1 ± 0.0

Mean ± SE;

* p < 0.05 different than Non-Ob;

† *p < 0.05 different than type 2 diabetic (T2D)*.

At 1,630 h, subjects reported to the lab ~5 h fasted. An intravenous catheter was placed into a forearm vein. Baseline blood samples began at ~1,740 h. At 1,800 h, subjects consumed a mixed meal providing 10 kcal/kg of body weight with 1 gram/kg of carbohydrate (target macronutrient composition: 40% Carbohydrate, 35% Fat, 25% Protein; Actual kcal and macronutrients for each group listed in Table 1). Carbohydrates were capped at 90 grams for 7 obese and 6 T2D subjects, except for 5 obese and 1 T2D who received >90 grams of carbohydrate prior to the decision to cap the total amount of carbohydrates for participants due to gastrointestinal distress. Following the meal, subjects sat quietly until bedtime. In the PMEX condition, subjects exercised at 55% of VO_2_ peak for 45 min on the treadmill at 2,000 h. Blood samples were collected at the following time points: −20, 0, 5, 10, 15, 20, 30, 40, 50, 60, and then every 15 min until the completion of the study. Blood was collected in EDTA tubes with and without DPP-IV inhibitor and aprotinin, placed on ice, centrifuged, aliquoted, and stored at −80°C until analysis. Blood samples were analyzed for glucose (YSI 2300 STAT PLUS, YSI Incorporated, Yellow Springs, OH), and insulin and glucagon concentrations (Human Metabolic Hormone Panel, Milliplex, Millipore Sigma), as well as other hormones not reported here. There was a minimum of 3 weeks between study days.

### Statistics and Data Analysis

For this study, we have defined low blood glucose as 70 mg/dL according to the American Diabetes Association (12, 13), but also examined the data below 50 and 60 mg/dL. Calculations were made for the Insulinogenic Index [insulin (30–0 min)/glucose (30–0 min)] and Matsuda Index (14). Baseline characteristics were analyzed by one-way ANOVA for statistical differences between groups. Repeated measure ANOVA (RMANOVA) tested interactions between study days (NOEX, PMEX), with a between-subject factor group (non-obese, obese, obese+T2D) and sex (Male, Female). Responses to the meal over time were tested with RMANOVA with study day by time (25 time points between −20 and 300 min) with a between-subject factors of group and sex. *Post-hoc* analysis were performed with a one-way ANOVA and Tukey comparison to explore group differences within dependent variables. Significance set at *P* < 0.05, and data are presented as mean ± SEM.

## Results

By design, the non-obese group was significantly lighter, had a lower percent body fat, and a lower BMI than the obese subjects with and without T2D (*P* < 0.05) (Table 1). The mean age for all subjects was 46.3 ± 2.1 yrs. Fasting glucose levels were in the normal range for both the non-obese and obese subjects. The non-obese subjects were more aerobically fit than the obese subjects with and without T2D (*P* < 0.05, Table 1), yet were still considered sedentary.

### Glucose

**Figure 1:**
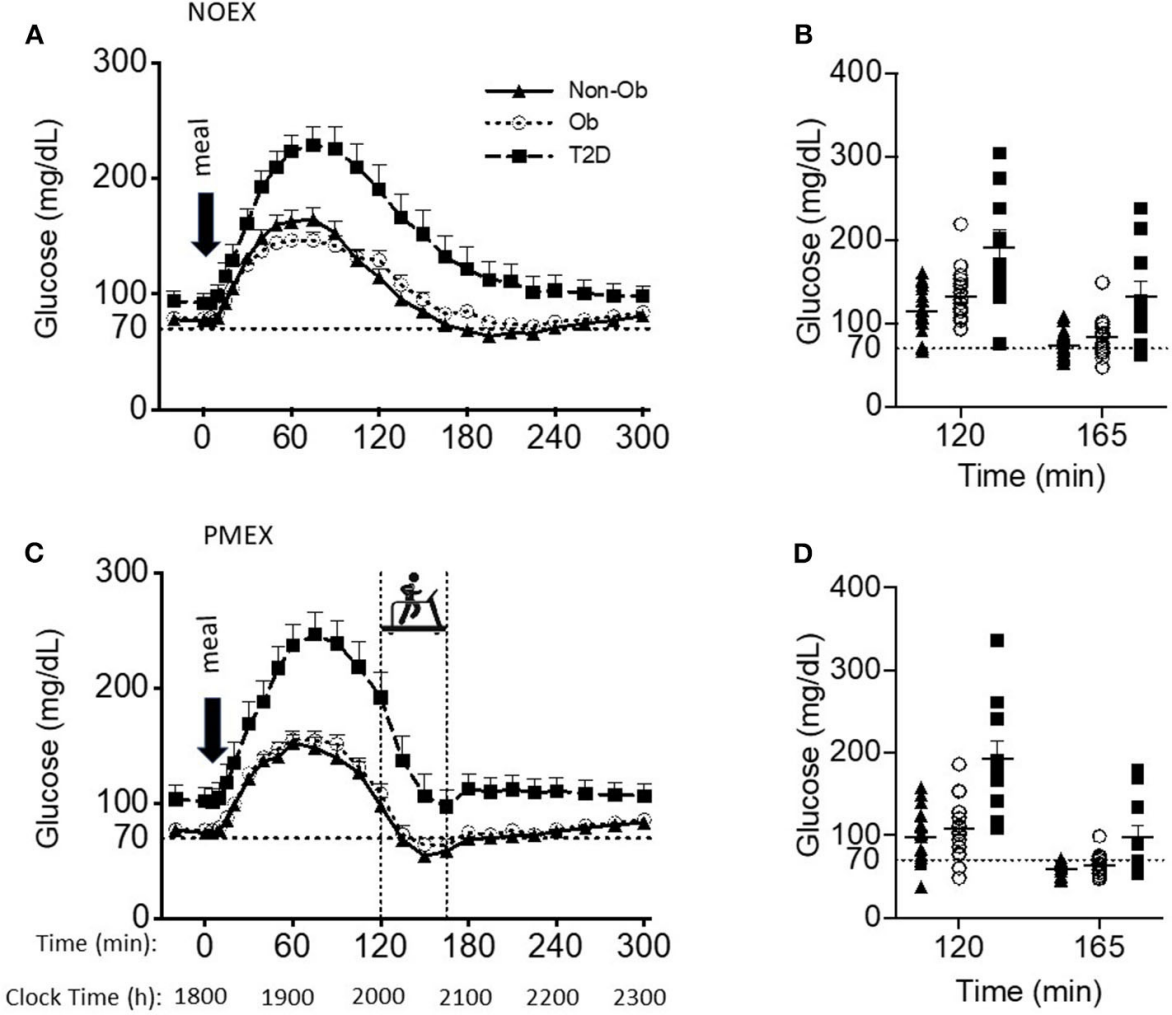
Glucose responses to a dinner meal in non-obese and obese individuals with and without type 2 diabetes (T2D) on a study day **(A)** with no post dinner exercise or a study day **(C)** with exercise (PMEX- 45 min, at 55%VO_2_ max). Individual glucose values at time point 120 and time point 165 in each group on the NOEX day **(B)** or PMEX day **(D)**. Mean ± SE.

Baseline glucose levels (~1,800 h) were similar between study days, but the individuals with T2D (99.4±6.6 mg/dL) had higher baseline glucose levels than the obese and non-obese subjects (79.8 ± 5.4 mg/dL; 76.6 ± 4.0 mg/dL, respectively, group effect: *P* < 0.05). The meal responses revealed a study day by time by group interaction (*P* < 0.001), such that during NOEX, glucose concentrations were elevated for T2D compared to non-obese adults from time −20 min until 15 min post meal consumption (*P* < 0.05), and from 30 to 300 min (*P* < 0.05) compared to both non-obese and obese subjects (Figure 1A). During PMEX in individuals with T2D, glucose concentrations were elevated compared to the obese and non-obese groups at all time points (*P* < 0.05, Figure 1C).

Peak glucose responses to the meal were similar between study days, but individuals with T2D had peak values of 248.5 ± 11.1 mg/dL, which was significantly greater than both obese and non-obese individuals (164.8 ± 8.7 mg/dL; 170.6 ± 8.2 mg/dL, respectively, *P* < 0.001). The time of the glucose peak was not different between study days or groups.

Glucose concentrations 2 h post meal consumption (start time of PMEX) were elevated during NOEX (145.9 ± 6.5 mg/dL) compared to PMEX (134.1 ± 6.7 mg/dL; *P* = 0.01, Figures 1B,D). Subjects with T2D had higher glucose concentrations (191.2 ± 12.7 mg/dL) at time 120 than both obese (122.6 ± 5.0 mg/dL) and non-obese subjects (106.2 ± 9.5 mg/dL; *P* < 0.001). Glucose concentrations at the start of exercise had a study day by group interaction (*P* < 0.01). *Post-hoc* analysis revealed that subjects with T2D had a greater drop in glucose concentration during PMEX (−94.8 ± 13.3 mg/dL) compared to both obese (−44.8 ± 7.1 mg/dL; *P* < 0.01) and non-obese (−39.3 ± 8.7 mg/dL; *P* < 0.01) subjects. Further examination of the change in glucose levels from the beginning to the end of exercise showed a trend for a study day by group by gender interaction (*P* = 0.08), where significant differences were observed by gender (*P* < 0.01). From time 120 to 165, glucose levels decreased more so in females than males in both conditions (NOEX, −55.0 ± 4.1 vs. −29.5 ± 6.8 mg/dL, *P* < 0.01; PMEX, −66.0 ± 6.3 vs. −38.9 ± 10.4 mg/dL, *P* = 0.03). Additionally, females with T2D had a larger drop in glucose levels from time 120 to 165 than males in both conditions (NOEX, −66.8 ± 7.8 vs. −23.5 ± 15.5 mg/dL, respectively, *P* = 0.02; PMEX, −109.9 ± 11.9 vs. −34.5 ± 23.7 mg/dL, respectively, *P* < 0.01).

The blood glucose nadir was different between study day and groups, with a nadir of 80.1 ± 5.0 mg/dL in subjects with T2D, 60.2 ± 4.0 mg/dL in obese subjects, and 53.3±3.7 mg/dL in non-obese subjects; T2D had a higher nadir than both other groups (*P* < 0.01, Figures 1B,D). However, hypoglycemia (<70 mg/dL) was prevalent amongst all study days for at least some individuals in all groups. During NOEX, 17 non-obese, 12 obese, and 4 T2D experienced hypoglycemia, while during PMEX, 17 non-obese, 17 obese, and 5 T2D experienced hypoglycemia. Collectively, 25 participants (15 Non-obese, 10 obese, and 2 obese+T2D) experienced hypoglycemia throughout both conditions. More severe hypoglycemia 60 mg/dL (and 50 mg/dL) was also noted in our subjects: during NOEX, 12 (3) non-obese, 7 (2) obese, and 2 (0) obese+T2D; during PMEX 16 (14) non-obese, 14 (6) obese, and 4 (1) obese+T2D. Only 2 obese+T2D were symptomatic, and this occurred at glucose levels above 70 mg/dL.

### Insulin

**Figure 2:**
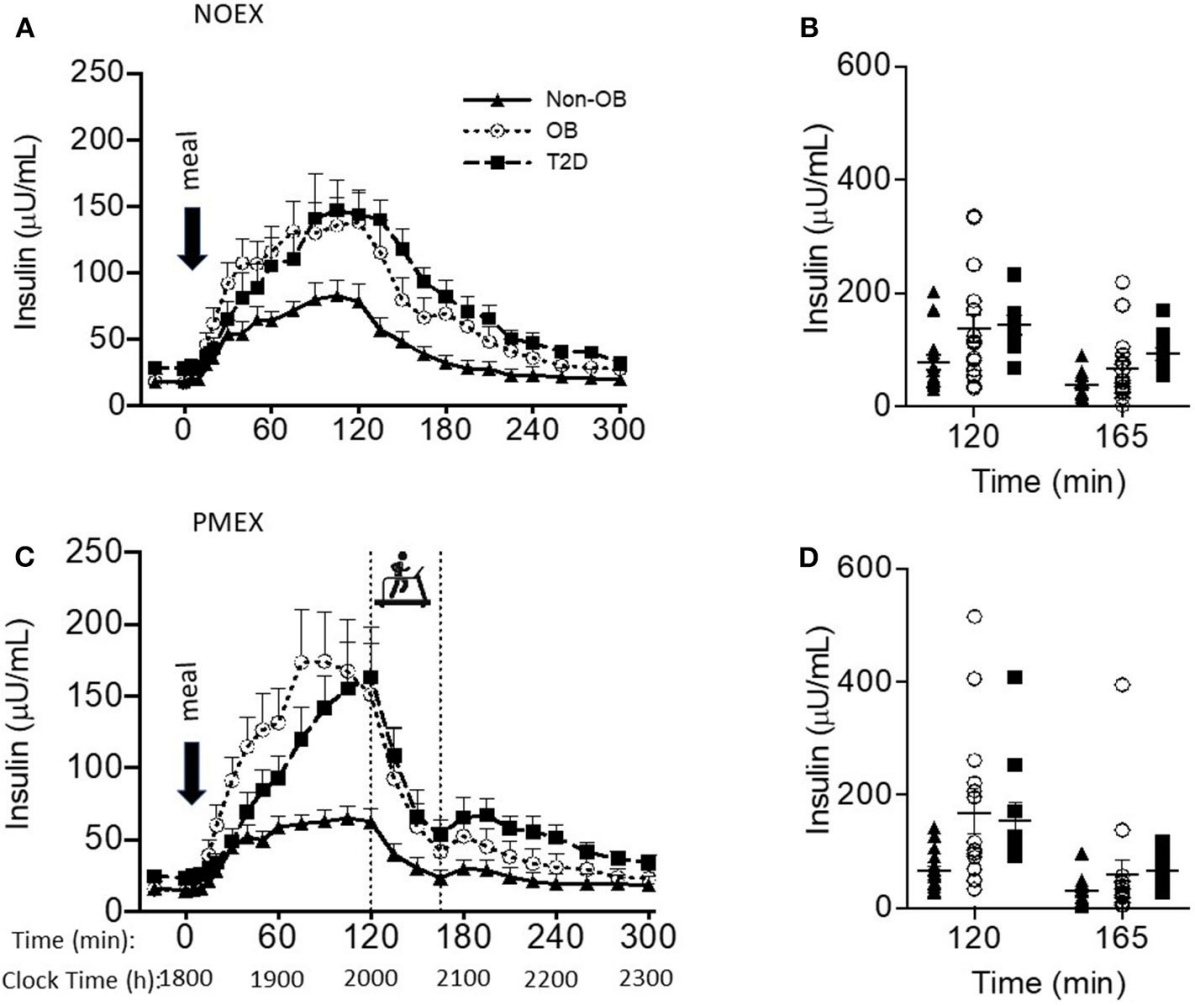
Insulin responses to a dinner meal in non-obese and obese individuals with and without type 2 diabetes (T2D) on a study day **(A)** with no post dinner exercise or a study day **(C)** with exercise (PMEX- 45 min, at 55%VO_2_ max). Individual glucose values at time point 120 and time point 165 in each group on the NOEX day **(B)** or PMEX day **(D)**. Mean ± SE.

Baseline insulin concentrations had a main effect of study day (*P* < 0.05), where NOEX was elevated compared to PMEX (*P* = 0.001). Insulin responses to the dinner meal had a study day by time by group interaction (*P* < 0.001), and *post-hoc* analysis revealed that individuals with T2D had elevated insulin concentrations compared to non-obese participants during NOEX from 135 to 225 min post meal consumption (*P* < 0.05). During NOEX, obese participants were elevated compared to non-obese at times 60, 75, and 135 (*P* < 0.05, Figure 2A). During PMEX, insulin concentrations in obese individuals were elevated compared to non-obese participants from 20 to 120 min post meal, and insulin concentrations in subjects with T2D were elevated compared to non-obese subjects at times 120, 195, 210, 225, and 240 (Figure 2C). Five hours after the dinner meal, insulin concentrations were similar between all groups in all conditions.

Peak insulin responses to the dinner meal were not different between study day, but non-obese participants had lower peak insulin concentrations (95.9 ± 21.9 μU/mL) compared to obese (187.2 ± 23.3 μU/mL; *P* < 0.01) and lower than T2D (183.8 ± 28.5 μU/mL; *P* = 0.02, Figure 2B). Time of peak insulin was not different between study days or groups.

Two hours post meal, insulin concentrations were not different by study day, but insulin concentrations were lower in non-obese subjects (70.6 ± 20.1 μU/mL) compared to both other groups (obese 145.5 ± 21.4μU/mL, *P* = 0.02; T2D 153.7 ± 26.3 μU/mL, *P* = 0.02). The change in insulin levels from the beginning of exercise (time 120) to the end of exercise (time 165) had a study day by group interaction (*P* = 0.03), such that non-obese individuals (39.2 ± 8.1 μU/mL) had less of a drop during PMEX compared to T2D (109.7 ± 30.1 μU/mL; *P* < 0.05) and tended to have less of a drop compared to obese (102.3 ± 22.6 μU/mL; *P* = 0.06). The insulin response to exercise had a gender by group interaction (*P* < 0.05), where obese males had a larger drop in insulin compared to obese females (160.8 ± 30.1 vs. 69.9 ± 14.5 μU/mL; *P* < 0.01). Nadir insulin concentrations were different by study day (*P* < 0.001) but not between subject group (Figures 2B,D). Participants experienced the lowest insulin concentrations during PMEX (18.8 ± 3.1 μU/mL) compared to NOEX (23.6 ± 3.4 μU/mL, *P* < 0.001).

### Insulin Sensitivity

Matsuda calculations were different between study day (*P* < 0.05) and group (*P* < 0.01). Non-obese participants had a higher Matsuda index than participants with T2D (7.8 ± 1.2 vs. 1.9 ± 1.5, respectively; *P* < 0.01); obese participants had a Matsuda index (5.3 ± 1.2) that was similar to the T2D. No gender differences were noted for the Matsuda index.

Insulinogenic index to the dinner meal was not different between study days but was significantly different between groups. Obese subject's insulinogenic index (1.6 ± 0.2) was highest compared to all other groups (non-obese, 0.6 ± 0.2; T2D, 0.5 ± 0.3; *P* < 0.01). We found no relationship between insulin sensitivity (Matsuda and insulin index) and the change in glucose levels during exercise, but the glucose nadir and insulin nadir during PMEX was negatively correlated with the Matsuda Index (*r* = −0.331, *P* < 0.05 and *r* = −0.476, *P* < 0.001, respectively).

The insulinogenic index was negatively correlated with the exercise-induced change in glucose on both study days (NOEX, *r* = −0.362, *P* < 0.05; PMEX, *r* = −0.399, *P* < 0.01) and positively with exercise-induced changes in insulin on both study days (NOEX, *r* = 0.586, *P* = 0.001; PMEX, *r* = 0.394, *P* < 0.03).

### Glucagon

**Figure 3:**
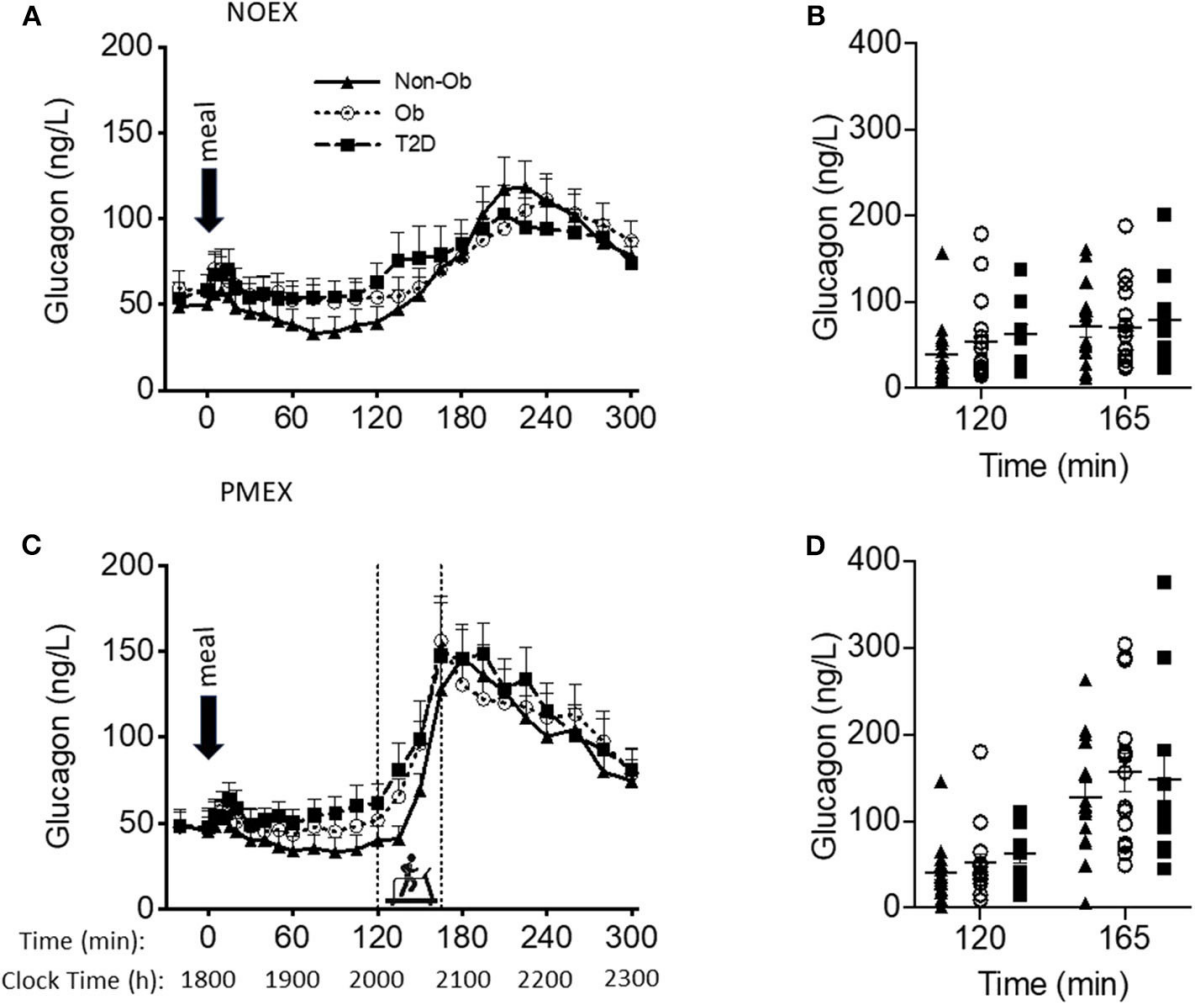
Glucagon responses to a dinner meal in non-obese and obese individuals with and without type 2 diabetes (T2D) on a study day **(A)** with no post dinner exercise or a study day **(C)** with exercise (PMEX- 45 min, at 55%VO_2_ max). Individual glucose values at time point 120 and time point 165 in each group on the NOEX day **(B)** or PMEX day **(D)**. Mean ± SE.

Baseline glucagon concentrations were different by study day (*P* < 0.05), with higher levels on the NOEX study day (53.4 ± 5.3 ng/L) compared to PMEX (47.7 ± 5.6 ng/L; *P* < 0.01). A group by gender interaction was shown for baseline glucagon concentrations (*P* < 0.05), where T2D males had higher concentrations compared to females (98.8 ± 22.7 ng/L vs. 40.4 ± 11.3 ng/L; *P* < 0.05). Although the response to the dinner meal had a study day by time interaction (*P* < 0.001), with NOEX having elevated glucagon levels compared to PMEX at times 0, 5, 10, 40, and 60, but lower than PMEX at times 150, 165, 180, 195, and 210 (*P* < 0.05 for all time points, Figures 3A,C). Glucagon concentrations were similar after 5 h. During PMEX, peak glucagon concentrations were elevated (180.1 ± 13.0 ng/L) compared to NOEX (126.2 ± 9.8 ng/L; *P* < 0.001). A gender by group interaction was also seen for peak glucagon concentrations with T2D males having higher peak values than females (257.5 ± 42.5 ng/L vs. 123.4 ± 21.2 ng/L; *P* < 0.01). The time of peak glucagon concentrations occurred earlier with PMEX (183.8 ± 5.6 min) than the NOEX condition (239.8 ± 5.4 min, *P* < 0.001) (Figures 3B,D). Additionally, obese males peak later than females (237.5 ± 12.9 min vs. 208.1 ± 6.5 min; gender by group interaction, *P* < 0.05).

Two hours post meal consumption, no differences in glucagon concentrations were observed by study days. In response to PMEX (time 120 to 165), glucagon levels increased by 95.2 ± 11.5 ng/L compared to NOEX at 22.3 ± 3.6 ng/L, *P* < 0.001. There was a study day by group by gender interaction (*P* = 0.02), such that male participants with T2D had an increased glucagon response during NOEX and PMEX compared to females (NOEX, 46.0 ± 15.5 vs. 8.2 ± 7.8 ng/L, *P* = 0.04; PMEX, 228.2 ± 45.0 vs. 50.4 ± 22.5 ng/L, *P* = 0.001).

## Discussion

This is one of the first studies to examine the occurrence of moderate-intensity exercise related hypoglycemia in untrained individuals following a dinner meal. We demonstrate here that in a group of relatively sedentary individuals, a dinner meal itself resulted in low glucose levels ~2 h post dinner, and in many individuals, there was a further exercise-induced decline, increasing the likelihood of hypoglycemia. This occurred in light of the fact that insulin concentrations were not exceedingly high prior to exercise, and glucagon levels were rising. Additionally, this large drop in glucose levels occurred in all subjects despite very different metabolic profiles. Upon completion of exercise, glucose concentrations in all subjects rebounded back to normoglycemic levels, an appropriate counterregulatory response. Further females with T2D experienced a larger drop in glucose levels with moderate-intensity exercise than seen in men.

It is well-known that exercise results in a dramatic increase in glucose turnover, which is a function of increased muscle utilization and increased production by the liver (15, 16), and this occurs in an intensity-dependent manner. If glucose production fails to stay abreast of the pace of glucose uptake, blood glucose concentrations fall. Changes in the hormonal milieu occur, with a fall in insulin levels, and increased catecholamines and glucagon levels in an effort to maintain blood glucose levels. An early study (17) showed that hypoglycemia occurs in moderately active men during prolonged exercise with an exaggerated rise in plasma epinephrine. Simultaneously others (18) have noted that maintaining glucoregulation during moderate-intensity exercise can result in decrements in insulin, increments in glucagon, or both may occur, particularly if catecholamine release is not adequate. Adrenergic means are primarily involved in the prevention of hypoglycemia during exercise. Although we did not measure catecholamines, our subjects showed appropriate hormonal responses during PMEX with a 67% decrease in insulin levels and ~3.8-fold increase in glucagon levels. Despite an appropriate hormonal response in our subjects, glucose levels declined considerably in most individuals. This is in agreement with an early study (19) that also showed decreases in glucose levels with low intensity walking and similarly demonstrated a 4-fold increase in glucagon levels. We had anticipated that the non-obese and obese subjects would not display the same degree of hypoglycemia as individuals with T2D, because we hypothesized they would have better counterregulatory control, due to their enhanced metabolic flexibility.

Surprisingly almost all of our non-obese and obese subjects had glucose levels below 70 mg/dL during moderate-intensity exercise, as well as 50% of the individuals with T2D. Even more noteworthy is that ~89% of the non-obese had glucose concentrations below 60 mg/dL, with 11 of them with low values on both study days, and 14 individuals with values below 50 mg/dL during exercise. Likewise, 39% of the obese subjects had glucose values below 60 mg/dL on the NOEX condition, and 78% on the PMEX condition. In individuals with T2D, 20% had glucose values below 60 mg/dL during NOEX, but 40% went below 60 mg/dL during PMEX. These low glucose levels have been reported before (4) but in response to prolonged exercise. Surprisingly only on two occasions did subjects become symptomatic, and in both conditions, this occurred in individuals with T2D in the absence of true hypoglycemia. This suggests that during exercise, there is not a distinct threshold for hypoglycemia and symptoms (20), and despite low circulating glucose levels, the brain must be sensing adequate glucose levels (20).

The susceptibility to hypoglycemia may be linked to the degree of insulin sensitivity of the individual. We found that insulin sensitivity (Matsuda) correlated modestly negative with the change in insulin during exercise. Further, we found no relationship between insulin sensitivity (Matsuda and insulin index) and the change in glucose levels during exercise, but the glucose nadir and insulin nadir during PMEX was negatively correlated the Matsuda Index indicating that the more insulin sensitive an individual was, the lower the glucose and insulin concentration went during exercise. This alludes that more insulin sensitive individuals may be more prone to these low glucose concentrations and remain asymptomatic despite very low glucose levels across adults of various glycemic control. It should also be considered that the lack of relationship with Matsuda may be due to the fact that insulin is not necessary for glucose disposal during exercise. However, Jentjens et al. found no relationship between hypoglycemia and insulin sensitivity (21). Recently, Kondo et al. (6) showed a transient exercise-induced hypoglycemia (at min 15 of exercise) in those individuals who were fasted overnight and ingested carbohydrates 30 min prior to exercise. These individuals had higher insulin concentrations at the start of exercise when compared to their counterparts that did not display hypoglycemia. Kuipers et al. (3) suggested that the occurrence of hypoglycemia may be due to a combination of enhanced insulin sensitivity, the quantity of glucose ingested, and low sympathetic activity.

Most of the previous reports on hypoglycemia have primarily focused on men. Earlier research has reported that blood glucose levels decline to a greater extent in premenopausal women than in men during prolonged fasting (22). Here we found that females demonstrated a greater drop in glucose levels 2–2.5 h post meal regardless of exercise. The females with T2D decreased glucose levels by 30.7% while the men decreased by 19.9% during rest and even larger decrease with exercise, 51.8% for females, and 32.0% for men. The smaller decrease in men may be attributed to a greater response in glucagon concentrations, thus maintaining their blood glucose levels. In response to exercise, T2D men had 4.5-fold higher glucagon responses during exercise compared to female T2D, with an even larger fold increase (5.6-fold) during the same time period but with rest. Potentially women with T2D may be more susceptible to hypoglycemia as glucose substantially drops 2 h after an evening meal without a compensatory glucagon response.

Human studies have shown circadian rhythms for glucose tolerance and energy metabolism, displaying enhanced glucose/meal tolerance in the morning over the nighttime hours (23). Much of the prior research examining transient hypoglycemia follows a 10 h overnight fast or with a small meal prior to exercise. Due to the circadian variations in glucose tolerance/insulin sensitivity, it may be possible that post dinner exercise may lower glucose levels more dramatically than seen in the morning hours. We have previously shown that glucose integrated area under the curve was reduced by ~18% when exercise preceded the dinner meal, while it decreased by ~30% when exercise was after the dinner meal (7). Considerably more research needs to be conducted examining the effect of exercise timing during the day, particularly in individuals with insulin resistance and/or impaired fasting glucose levels.

Most studies have focused on exercise-induced hypoglycemia in the early morning period after prolonged fasting or having a small pre-exercise meal (3, 5, 6, 21). Further, much of this research only studied young healthy men, thus a strength of this project is that both men and women were included, subjects were sedentary to moderately active, and female participants were both pre- and post-menopausal. An additional strength of this study is the large sample size, which included individuals with varying degrees of insulin sensitivity. A limitation of this study is that unlike previous work, this study only examined the glucose response beyond the evening meal and did not compare to a similar meal earlier in the day, thus it is unclear if we would have seen the same degree of hypoglycemia following a breakfast meal. However, we do not think this could explain our higher incidence of hypoglycemia as Van Cauter et al. (24) reported declines in the glucose tolerance from morning to evening in healthy individuals.

In conclusion, moderate-intensity post-meal exercise in the evening results in transient hypoglycemia in many individuals across a spectrum of glycemic status. More work needs to be conducted examining exercise at different times of the day to establish if the same phenomena is occurring in the morning or early afternoon.

## Data Availability Statement

The raw data supporting the conclusions of this article will be made available by the authors, without undue reservation.

## Ethics Statement

The studies involving human participants were reviewed and approved by University of Missouri Institutional Review Board. The patients/participants provided their written informed consent to participate in this study.

## Author's Note

Exercise-induced hypoglycemia has primarily been investigated by the effects on performance by examining meal timing, quantity of carbohydrate, and fasting vs. fed states, in an attempt to establish the best protocol to minimize hypoglycemia. What is unclear is whether moderate-intensity exercise causes hypoglycemia, and is it related to metabolic health, such as insulin sensitivity. In this study, 46 non-obese, obese and obese+type 2 diabetes (T2D) men and women completed two conditions: (1) in the evening, following a dinner meal over 5 h (NOEX) and (2) preforming 45 min of moderate-intensity exercise 2 h post dinner meal (PMEX). We observed a similar glucose, insulin, and glucagon response to the meal on both occasions, with obese+T2D having the highest glucose responses. Glucose levels 70 mg/dL was prevalent in the evenings of both conditions, with 33 subjects during NOEX and 39 during PMEX. Severe hypoglycemia (50 mg/dL) was also seen, with 5 subjects during NOEX and 21 during PMEX (all but on 2 occasions were asymptomatic). The postmeal glucose decline was greater in females than males. In conclusion, a dinner meal can result in low glucose levels but moderate-intensity exercise can cause hypoglycemia in some individuals, which is usually asymptomatic.

## Author Contributions

All authors listed have made a substantial, direct and intellectual contribution to the work, and approved it for publication.

## Conflict of Interest

The authors declare that the research was conducted in the absence of any commercial or financial relationships that could be construed as a potential conflict of interest.
